# Semantic Memory Organization in Japanese Patients With Schizophrenia Examined With Category Fluency

**DOI:** 10.3389/fpsyt.2018.00087

**Published:** 2018-03-21

**Authors:** Chika Sumiyoshi, Haruo Fujino, Tomiki Sumiyoshi, Yuka Yasuda, Hidenaga Yamamori, Michiko Fujimoto, Ryota Hashimoto

**Affiliations:** ^1^Faculty of Human Development and Culture, Fukushima University, Fukushima, Japan; ^2^Department of Special Needs Education, Oita University, Oita, Japan; ^3^Department of Clinical Epidemiology, Translational Medical Center, National Center of Neurology and Psychiatry, Kodaira, Tokyo, Japan; ^4^Department of Psychiatry, Osaka University Graduate School of Medicine, Suita, Osaka, Japan; ^5^Molecular Research Center for Children’s Mental Development, United Graduate School of Child Development, Osaka University, Suita, Osaka, Japan

**Keywords:** schizophrenia, cognition, semantic memory, category fluency, singular value decomposition analysis

## Abstract

**Background:**

Disorganization of semantic memory in patients with schizophrenia has been studied by referring to their category fluency performance. Recently, data-mining techniques such as singular value decomposition (SVD) analysis have been reported to be effective in elucidating the latent semantic memory structure in patients with schizophrenia. The aim of this study is to investigate semantic memory organization in patients with schizophrenia using a novel method based on data-mining approach.

**Method:**

Category fluency data were collected from 181 patients with schizophrenia and 335 healthy controls at the Department of Psychiatry, Osaka University. The 20 most frequently reported animals were chosen for SVD analysis. In the two-dimensional (2D) solution, item vectors (i.e., animal names) were plotted in the 2D space of each group. In the six-dimensional (6D) solution, inter-item similarities (i.e., cosines) were calculated among items. Cosine charts were also created for the six most frequent items to show the similarities to other animal items.

**Results:**

In the 2D spatial representation, the six most frequent items were grouped in the same clusters (i.e., *dog, cat* as pet cluster, *lion, tiger* as wild/carnivorous cluster, and *elephant, giraffe* as wild/herbivorous cluster) for patients and healthy adults. As for 6D spatial cosines, the correlations (Pearson’s *r*) between 17 items commonly generated in the two groups were moderately high. However, cosine charts created for the three pairs from the six most frequent animals (*dog*–*cat, lion*–*tiger, elephant*–*giraffe*) showed that pair-wise similarities between other animals were less salient in patients with schizophrenia.

**Discussion:**

Semantic memory organization in patients with schizophrenia, revealed by SVD analysis, did not appear to be seriously impaired in the 2D space representation, maintaining a clustering structure similar to that in healthy controls for common animals. However, the coherence of those animals was less salient in 6D space, lacking pair-wise similarities to other members of the animal category. These results suggests subtle but structural differences between the two groups. A data-mining approach by means of SVD analysis seems to be effective in evaluating semantic memory in patients with schizophrenia, providing both a visual representation and an objective measure of the structural alterations.

## Introduction

Cognitive impairment in patients with schizophrenia is a cardinal feature of the disease and is generally independent of positive or negative psychiatric symptoms (e.g., hallucinations or withdrawal). This impairment disturbs favorable functional outcomes of patients, including daily living skills, social functioning, and work (1–4). Accordingly, comprehensive cognitive batteries have been developed to assess the cognitive function of patients with schizophrenia. Currently, the Brief Assessment of Cognition in Schizophrenia (BACS) (5) and MATRICS Consensus Cognitive Battery (MCCB) (6) are the most acknowledged batteries, and they have been used for research and clinical purposes.

Although those “gold-standard” cognitive batteries have been reported to be effective for predicting functional outcomes in patients with schizophrenia (7), the target domains are mainly executive aspects of cognition (i.e., attention, processing speed, and visual/verbal working memory). Higher order cognition, such as semantic memory, has received less attention, although disorganization of semantic memory has been considered as one of the intermediate cognitive phenotypes in patients with schizophrenia (8).

The paucity of studies seems to be largely due to the lack of powerful tools, such as the MCCB or BACS. For healthy subjects, cognitive experiments (e.g., semantic priming) have been frequently used to estimate the latent structure of semantic memory. However, an experimental setting is often too demanding for patients with mental disorders that attenuate attention or motivation.

Alternative methods have been developed to assess semantic memory in patients with schizophrenia. The aim of this study was to investigate semantic memory organization in patients with schizophrenia introducing a novel method based on data-mining approach. Earlier attempts in this line of research were also briefly reviewed.

## Previous Approach for Assessing Semantic Memory in Patients with Psychiatric Disorders

Less demanding methods, compared to experimental settings, have been explored for evaluating semantic memory organization in patients with schizophrenia. Most of them utilized verbal outputs in the category fluency task (CFT), partly because the CFT is included in established cognitive batteries (e.g., the MCCB and BACS), and also because the task is simple both for testers and subjects. The CFT is a free recall task, asking a subject to produce as many items in a given category (e.g., animal) as possible in a designated time (typically 1 min).

There are two lines of research on the methods for estimating semantic structures using the CFT. They differ in terms of measurement of similarities; one uses on “adjacency” while another uses “co-occurrence” of outputs in the CFT.

**Figure 1:**
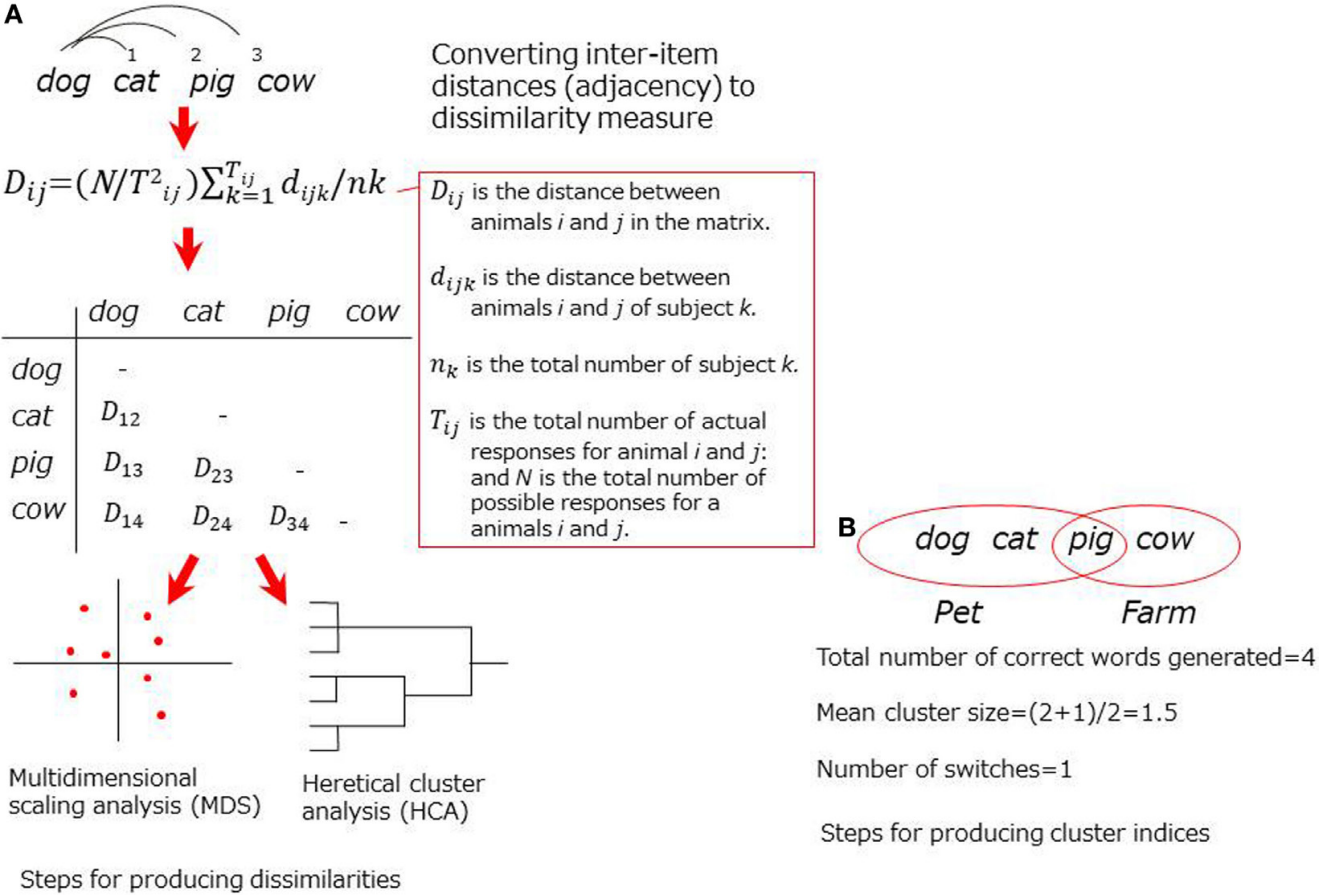
Schematic representation for adjacency-based techniques. **(A)** Steps for producing dissimilarities. **(B)** Steps for producing cluster indices.

The earlier approach focus on adjacency of the words produced in the CFT, assuming that it reflects semantic associations in memory. In some studies, specific formulas were modeled to convert the word order to dissimilarities (9–11) for submission to advanced statistical analyses to visualize the structures [e.g., multidimensional scaling (MDS) or hierarchical cluster analysis (HCA) (Figure 1)]. In another technique, cluster indices (i.e., a cluster size or a switching score) are designated based on predefined clustering rules (12). Studies using either technique have successfully demonstrated aberrant structurers of semantic memory in patients with schizophrenia (10, 12–15).

Critical limitations for adjacency-based approach, as noted above, have been addressed. In studies using formulas for dissimilarities, the results were likely to be inaccurate if the sample size was small (16, 17). In studies using cluster indices, scoring tended to be arbitrary because the predefined clustering rules (e.g., farm animals, pet) were somewhat intuitive. In addition, the clustering rules may not be universal across cultures (e.g., *pig* was listed in a pet cluster, but it may not be true in other countries like Japan).

## New Approach to Estimate Semantic Memory

**Figure 2:**
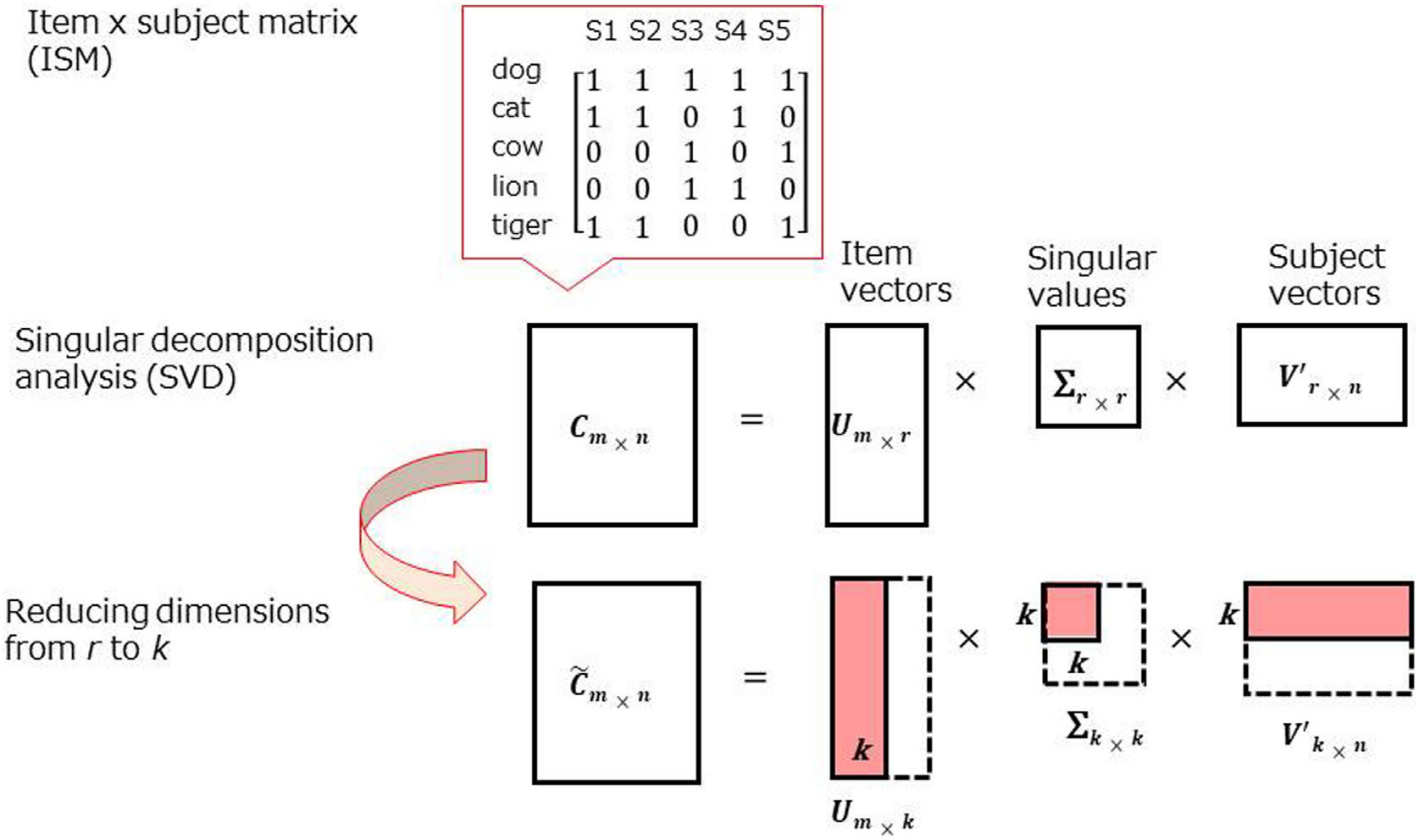
Schematic representation for singular value decomposition (SVD) analysis.

Recently, data-mining techniques, such as singular value decomposition (SVD), have been applied to the CFT to examine the deeper structure of semantic memory (18–20). SVD is a general matrix factorization technique based on eigenvalue decomposition [Figure 2; for further information, see supplementary materials in Ref. (18–20)].

One notable difference between the data-mining approach and adjacency-based techniques is the basic measurement with “co-occurrence” of items across the participants rather than “adjacency,” the latter of which has been used in previous techniques. For example, in the earlier techniques, *dog* and *cat* show higher proximity in a *dog, cat, pig* sequence than *dog, pig, cat*. In the new technique, the proximity would be the same as long as *dog* and *cat* are produced in a sequence of word outputs (i.e., “co-occur”) by a subject.

A strength of SVD analysis is that inter-item similarities can be estimated even if no subjects produce a particular pair (i.e., *dog*–*snake*), which can occur in small samples. Mathematical simplicity and clarity are also superior in SVD analysis. This established mathematical method has been used in many scientific fields, including genetics (21) as well as applied linguistics (22). In contrast, in adjacency-based techniques (9–11), formulas are presented to general high-end users without sufficient information for modeling.

Singular value decomposition analysis has already been applied to the CFT performance of patients with schizophrenia. Sung et al. (18) demonstrated subtle differences between patients with schizophrenia and healthy adults by looking at higher dimensional structures of semantic memory, which may not have been elucidated in studies using the previous techniques. In brief, patients with schizophrenia showed similar semantic clustering in the lower dimensional SVD solution, but it was less coherent in the higher dimensional solution, suggesting that semantic deterioration occurred in the latent structure.

## Preliminary Study

Given a positive result from a previous study using SVD analysis (18), we aimed to investigate semantic structures in Japanese patients with schizophrenia by applying SVD analysis to the CFT. In particular, we were interested in whether this novel method could also be useful to show structural differences in semantic memory between Japanese patients with schizophrenia and healthy adults as has been reported in previous studies (18).

### Participants

Data were collected from 181 patients with schizophrenia and 335 healthy controls at the Department of Psychiatry, Osaka University. Table 1 presents the characteristics of the participants. All patients met the DSM-IV criteria for schizophrenia (23). The diagnosis was made by experienced psychiatrists based on the Structured Clinical Interview for DSM-IV (SCID) for schizophrenia. Healthy controls were recruited from the community through local advertisements at Osaka University. All participants provided written informed consents. The study protocol was approved by the Ethical Committee of Osaka University, and the procedures were conducted according to the Declaration of Helsinki.

**Table 1:** Characteristics of participants.

	HC	SCZ	*x^2^/t*	*df*	*p*	*g*^e^
*N*^a^	335 (154/181)	181 (107/74)	8.12^b^	1	0.004	
Age	35.80 (11.90)	36.76 (12.16)	−0.87	514	0.383	−0.08
Education (years)	15.20 (2.20)	14.20 (2.49)	4.40	514	<0.0001	0.19
Duration (years)	–	12.66 (10.46)	–	–	–	–
Onset	–	24.10 (8.80)	–	–	–	–
Neuroleptics (mg)^c^	–	182.65 (365.76)	–	–	–	–
PANSS positive	–	14.79 (4.93)	–	–	–	–
Negative	–	17.48 (6.35)	–	–	–	–
Cognition	–	11.87 (4.12)	–	–	–	–
Excitement	–	8.23 (3.22)	–	–	–	–
Depression/anxiety	–	9.88 (3.53)	–	–	–	–
Full IQ	108.67 (12.28)	86.93 (17.56)	15.68	479	<0.0001	1.55
Performance IQ	109.31 (12.15)	91.76 (16.94)	11.98	437	<0.0001	1.21
Verbal IQ	107.13 (13.11)	83.53 (17.08)	16.60	437	<0.0001	1.56
Premorbid IQ (JART)	107.09 (8.02)	101.48 (10.17)	6.88	514	<0.0001	0.51
LFT score^d^	10. 07 (2.96)	7.43 (2.75)	9.90	514	<0.0001	0.91
CFT score	20.94 (4.51)	15.86 (4.67)	12.06	514	<0.0001	0.69

*HC, healthy controls; SCZ, patients with schizophrenia; PANSS, The positive and negative syndrome scale; JART, Japanese Adult Reading Test; LFT, Letter fluency task; CFT, Category fluency task*.

*Male/female and SD are presented in parentheses*.

*^a^Several variables had missing values. Degree of freedom varied accordingly*.

*^b^Chi-squared test*.

*^c^CPZ equivalent*.

*^d^The mean of the three letters*.

*^e^Hedges’s *g* (effect size)*.

### Assessment

#### Verbal Fluency Tasks

The CFT and letter fluency task (LFT) were administered following the normative method (24). In the CFT, an animal was used as a cue, while three hiragana letters (“*fu*,” “*a*,” and “*ni*”) were used in the LFT. Subjects were asked to produce as many animal names (CFT) or words beginning with a specified letter (LFT) as possible in one minute. The CFT score represented the total outputs for animal category, while the LFT score represented the mean of outputs for three letters. Errors [i.e., repetitions, proper nouns, and intrusions (e.g., *apple* for an animal cue)] were excluded from outputs.

#### Intelligence

Current intelligence (full-scale intelligence quotient, FIQ) was assessed by the Japanese version of the Wechsler Memory Scale-Third edition (WAIS-3) (25) as part of a larger neuropsychological assessment (26–30). The third edition was used because the fourth edition has not yet been released in Japan. Premorbid intelligence was estimated by the Japanese version of the Adult Reading Test (JART) (31). This test is composed of 50 Japanese kanjis (ideographic scripts), and the reading task is considered to be equivalent to irregular word reading employed in the National Adult Reading Test (31–33).

#### Psychiatric Symptoms

The patients were assessed with the Positive and Negative Syndrome Scales (34) to evaluate psychiatric symptoms. The evaluation was made following the five-factor model of the scale (i.e., positive, negative, cognition, excitement, and depression/anxiety) (35, 36).

### Analysis

#### Characteristics of Participants

Male-female ratio was tested by *x2* test. Other demographic characteristics (age and years of education), IQ measures (FIQ and JART), and verbal fluency measures (CFT score and LFT score) were compared between patients and healthy controls using *t*-tests. In addition, effects sizes (Hedges’s *g*) were calculated for relevant variables. The statistical significance was set at *p* < 0.05 (two-tailed) in all analyses. SPSS ver. 22.0 was used for statistical analyses.

#### SVD Analysis

As noted in Assessment section, rule breaks (i.e., repetitions, intrusions, and proper nouns) were removed from the analysis. An item × subject matrix (ISM) was created for the patient group and healthy adult group (two matrices in total). Rows of the ISM contained animal items (e.g., *dog, cat*, etc.), while columns contained subjects, and each cell contained a co-occurrence of items (Figure 2, top). Each row (i.e., item) is treated as a vector in the space produced by SVD. Due to technical limitations in creating large-scaled ISMs, 20 of the most frequently reported animals in each group were chosen for SVD analysis (Table 2, above the line).

**Table 2:** Frequency ranks of animal items.

Rank	HC (*N* = 335)	Frequency	SCZ (*N* = 181)	Frequency
1	Dog	309	Dog	169
2	Cat	305	Cat	163
3	Lion	250	Lion	143
4	Giraffe	244	Elephant	119
5	Tiger	239	Giraffe	119
6	Elephant	235	Tiger	116
7	Monkey	234	Monkey	106
8	Horse	171	Cow	74
9	Sheep	163	Horse	74
10	Cow	155	Mouse	69
11	Mouse	152	Rabbit	64
12	Rabbit	148	Hippopotamus	63
13	Hippopotamus	143	Bird	62
14	Bear	122	Sheep	62
15	Rhinoceros	116	Pig	55
16	Bird	115	Bear	53
17	Panda	110	Leopard	49
18	Cheetah	102	Deer	46
19	Snake	102	Snake	43
20	Zebra	102	Zebra	40

21	Wildboar	101	Rhinoceros	39
22	Gorilla	96	Panda	37
23	Leopard	95	Wildboar	36
24	Whale	92	Fox	35
25	Koala	87	Cheater	32
26	Dolphin	83	Seaotter	32
27	Penguin	83	Whale	32
28	Chimpanzee	77	Goat	29
29	Deer	76	Squirrel	29
30	Orangutan	71	Dolphin	28
31	Pig	69	Raccoondog	28
32	Goat	68	Gorilla	27
33	Racoondog	68	Chimpanzee	25
34	Fox	67	Crocodile	25
35	Hen	65	Koala	25
36	Kangaroo	65	Hen	24
37	Sparrow	61	Penguin	24
38	Crocodile	56	Sparrow	24
39	Camel	52	Pigeon	23
40	Seaotter	52	Crow	21

*HC, healthy controls, SCZ, patients with schizophrenia*.

*The most frequent 20 items were submitted to singular value decomposition analyses*.

Item vectors in reduced dimensions were used to produce a visual representation and inter-item similarities. For visual interpretation, item vectors were plotted on the two-dimensional (2D) space, while inter-item similarities were calculated in a higher dimensional space. In SVD analysis, inter-item similarities were presented by the cosines between item vectors in SVD analysis, but not the Euclidian distance between items as presented in the MDS analysis. Accordingly, a cosine close to 1.0 indicates that two items are highly similar (two words frequently co-occur across subjects), while −1.0 implies that they are most dissimilar (two words are produced independently).

R ver. 3.2.2 (37) and its LSA package (38) were used for conducting SVD analysis and producing inter-item cosines.

### Results

#### Group Comparisons

Table 1 presents results from group comparisons. The verbal fluency performance was significantly better in healthy controls than patients with schizophrenia (LFT score: *t* = 9.90, df = 514, *p* < 0.001, CFT: *t* = 12.06, df = 514, *p* < 0.001). The same trend was found in intelligence measures (FIQ: *t* = 15.68, df = 479, *p* < 0.001, VIQ: *t* = 11.98, df = 437, *p* < 0.001 PIQ: *t* = 16.60, df = 437, *p* < 0.001, premorbid IQ: *t* = 6.88, df = 514, *p* < 0.001). Age did not significantly differ between the two groups (*t* = −0.87, df = 514, *p* = 0.38). Patients had less education than healthy adults (*t* = 4.40, df = 514, *p* = 0.001) although the difference was small as was indicate by the minor effect size (*g* = 0.19).

#### SVD Analysis

As previous studies have suggested (39), there is no statistical rules for choosing an appropriate number of singular values (dimensions) for the dimensionality reduction. Therefore, the number was determined at the point at which a fraction of the sum of the selected singular values to the sum of all singular values reached 0.5. A six-dimensional solution (6D) satisfied the criterion, and therefore, inter-item cosines were calculated in this dimension. As noted earlier, a 2D solution was also produced in which item vectors were plotted on the 2D space.

##### Two-Dimensional Space Representations

**Figure 3:**
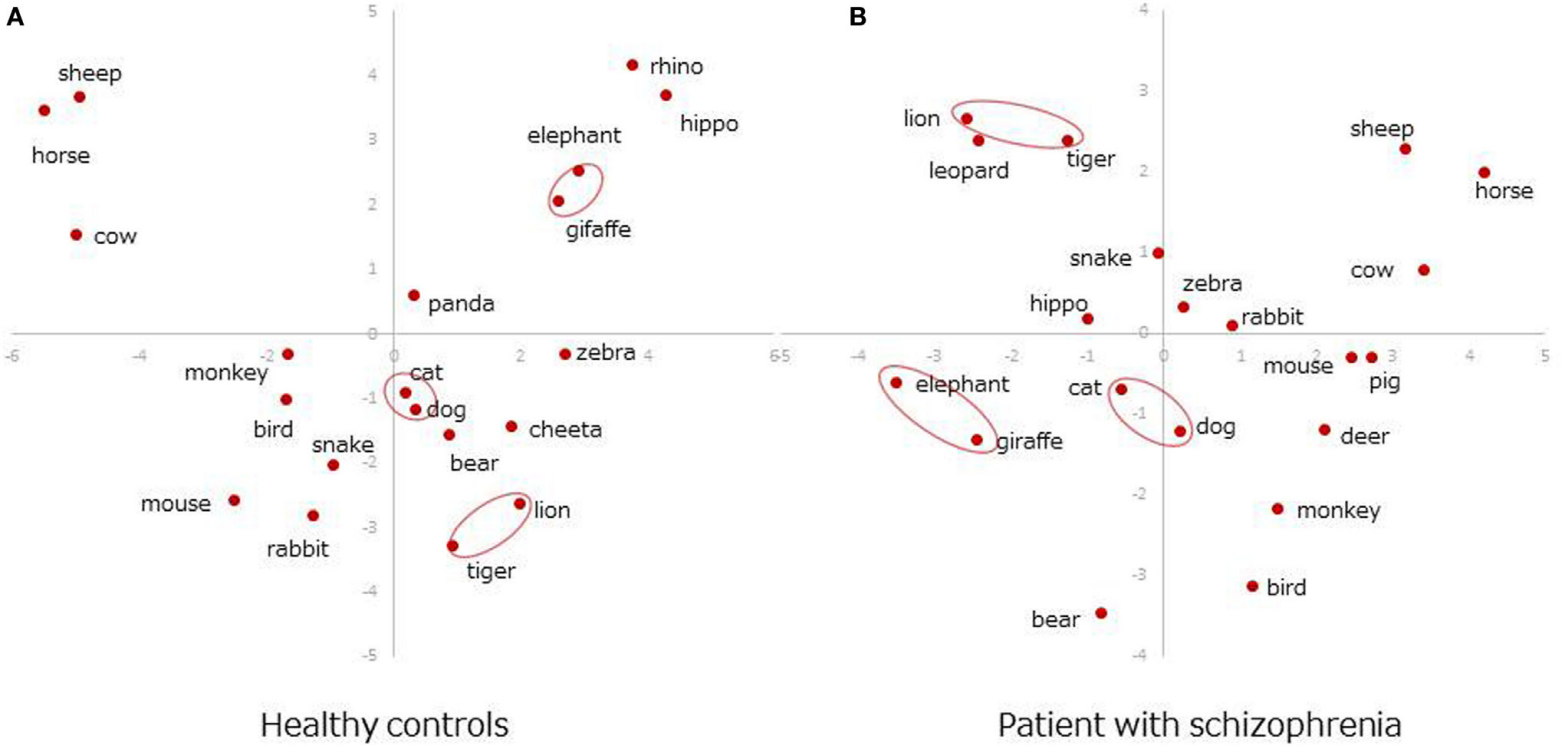
Two-dimensional space representations. **(A)** Healthy controls. **(B)** Patient with schizophrenia.

Figure 3 presents the plots of the most frequently produced 20 items (Table 2) on 2D space. Dimensions 2 and 3 were used because the first dimension in SVD solutions is generally determined by the frequencies of items in the whole dataset, and it is not informative for showing semantic associations (18). Overall, the most frequent six items were grouped in the same clusters between patients and healthy adults: *dog, cat* as a pet cluster, *lion, tiger* as a wild/carnivorous cluster, and *elephant, giraffe* as a wild/herbivorous cluster (Figures 3A,B, circled items).

##### Cosines in Six-Dimensional Space

Table 3 shows the inter-item cosines of the 20 most frequent items in healthy adults (Table 3A) and patients (Table 3B). Cosine values were all positive probably because only the 20 most frequent items were used. Due to high frequency, those items necessarily co-occurred with each other; therefore, the cosine values tended to be non-negative. Similar trends were found in a previous study [see Figure 2 in Ref. (18)], where cosines of highly frequent items (e.g., cat) yielded almost all positive values to other items. In that study, negative values appeared as the item became less frequent (e.g., whale).

**Table 3:** Cosines in six-dimensional space for the most frequent 20 items.

A. Healthy controls																			
	Bear	Bird	Cat	Cow	Dog	Elephant	Giraffe	Hippopotamus	Horse	Lion	Monkey	Mouse	Rabbit	Sheep	Snake	Tiger	Zebra	Deer	Leopard
Bear	0.47	0.71	0.58	0.71	0.69	0.79	0.51	0.55	0.70	0.56	0.59	0.62	0.43	0.56	0.71	0.52	0.33	0.39	0.95
Bird		0.78	0.55	0.79	0.56	0.58	0.52	0.64	0.69	0.82	0.49	0.37	0.67	0.82	0.67	0.32	0.58	0.41	0.49
Cat			0.80	1.00	0.93	0.93	0.80	0.80	0.96	0.96	0.84	0.75	0.81	0.76	0.95	0.81	0.72	0.73	0.67
Cow				0.79	0.75	0.74	0.49	0.97	0.74	0.78	0.87	0.65	0.94	0.46	0.77	0.56	0.50	0.47	0.62
Dog					0.92	0.93	0.80	0.79	0.96	0.96	0.83	0.74	0.80	0.77	0.95	0.80	0.72	0.72	0.67
Elephant						0.98	0.92	0.75	0.91	0.85	0.76	0.70	0.75	0.56	0.88	0.90	0.66	0.89	0.68
Giraffe							0.89	0.75	0.89	0.85	0.74	0.75	0.73	0.64	0.86	0.85	0.57	0.84	0.76
Hippopotamus								0.56	0.75	0.75	0.46	0.49	0.61	0.52	0.69	0.80	0.57	0.98	0.52
Horse									0.69	0.81	0.76	0.58	0.98	0.52	0.70	0.49	0.44	0.54	0.63
Lion										0.88	0.86	0.68	0.69	0.62	0.99	0.87	0.84	0.68	0.65
Monkey											0.80	0.76	0.85	0.83	0.87	0.72	0.63	0.69	0.50
Mouse												0.83	0.75	0.53	0.90	0.77	0.63	0.43	0.49
Rabbit													0.56	0.72	0.71	0.70	0.25	0.42	0.42
Sheep														0.54	0.70	0.52	0.49	0.60	0.51
Snake															0.61	0.40	0.27	0.38	0.42
Tiger																0.85	0.83	0.62	0.64
Zebra																	0.72	0.80	0.42
Cheetah																		0.56	0.35
Rhinoceros																			0.43

**B. Patients with schizophrenia**																			

	**Bear**	**Bird**	**Cat**	**Cow**	**Dog**	**Elephant**	**Giraffe**	**Hippopotamus**	**Horse**	**Lion**	**Monkey**	**Mouse**	**Rabbit**	**Sheep**	**Snake**	**Tiger**	**Zebra**	**Deer**	**Leopard**

Bear	0.58	0.62	0.25	0.62	0.49	0.59	0.55	0.31	0.54	0.80	0.53	0.49	0.29	0.19	0.53	0.33	0.62	0.37	0.09
Bird		0.59	0.66	0.66	0.59	0.51	0.12	0.35	0.57	0.71	0.63	0.51	0.27	0.58	0.61	0.58	0.66	0.26	0.69
Cat			0.75	0.99	0.91	0.97	0.76	0.73	0.86	0.85	0.68	0.71	0.72	0.82	0.85	0.91	0.76	0.68	0.66
Cow				0.80	0.63	0.61	0.46	0.80	0.63	0.64	0.86	0.80	0.75	0.77	0.69	0.90	0.70	0.45	0.88
Dog					0.91	0.95	0.71	0.74	0.86	0.88	0.72	0.73	0.71	0.84	0.85	0.92	0.80	0.64	0.72
Elephant						0.96	0.68	0.44	0.84	0.66	0.54	0.64	0.42	0.84	0.81	0.88	0.50	0.74	0.61
Giraffe							0.76	0.56	0.81	0.76	0.53	0.61	0.55	0.79	0.77	0.85	0.64	0.65	0.58
Hippopotamus								0.46	0.58	0.48	0.65	0.79	0.48	0.38	0.56	0.69	0.34	0.69	0.17
Horse									0.62	0.75	0.64	0.54	0.99	0.67	0.66	0.69	0.84	0.38	0.67
Lion										0.79	0.58	0.59	0.64	0.85	0.99	0.77	0.60	0.87	0.50
Monkey											0.63	0.53	0.72	0.66	0.80	0.64	0.93	0.46	0.57
Mouse												0.96	0.59	0.47	0.66	0.77	0.59	0.53	0.55
Rabbit													0.51	0.47	0.65	0.81	0.44	0.64	0.47
Sheep														0.66	0.67	0.66	0.79	0.43	0.59
Snake															0.85	0.86	0.61	0.65	0.81
Tiger																0.78	0.61	0.87	0.53
Zebra																	0.60	0.66	0.80
Deer																		0.19	0.70
Leopard																			0.19

**Figure 4:**
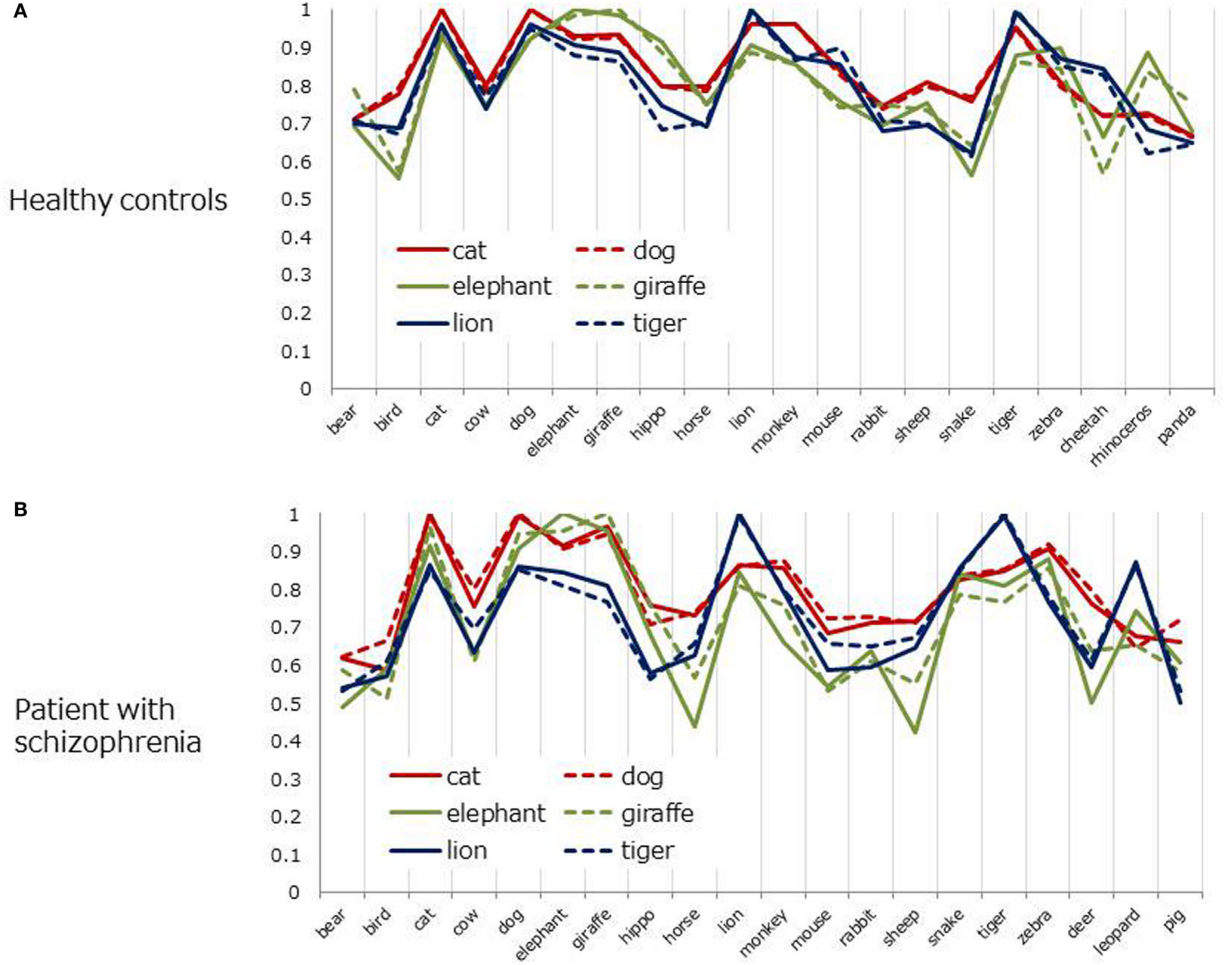
Cosine values between six frequent animals and other items (six-dimensional space). **(A)** Healthy controls. **(B)** Patient with schizophrenia.

*Items in gray columns are presented as line charts in Figure 4*.

Of all the 20 items, 17 items were in common between healthy adults and patients (i.e., *bear, bird, cat, cow, dog, elephant, giraffe, hippopotamus, horse, lion, monkey, mouse, rabbit, sheep, snake*, and *tiger*). Thus, the correlation (Pearson’s *r*) was calculated using those items to examine whether the cosine values were similar between the two groups. The correlation was moderately high (*r* = 0.78, *p* < 0.01), suggesting that a pattern of inter-item similarities between frequent items in patients with schizophrenia is comparable to that in healthy adults.

To further examine structural similarities (or differences) between the two groups, cosine charts (Figures 4A,B) were created for the six most frequent animals (i.e., d*og, cat, lion, tiger, elephant*, and *giraffe*). The lines represent 6D cosine values between a particular animal (e.g., *dog*) and the other most frequent 20 items. Overall, cosine values fluctuated more in patients than in healthy controls. In healthy controls, the patterns of line charts were highly similar between *dog*–*cat* pair (red, pet items) and the rest of the items. Similar trends were also found for the *lion*–*tiger* pair (blue, wild/carnivorous items) and *elephant*–*giraffe* pair (green, wild/herbivorous items) (Figure 4A). However, those pair-wise similarities were less salient in patients with schizophrenia, except for the *dog*–*cat* pair (Figure 4B).

## Discussion

We, first, reviewed the methods to evaluate semantic memory organization in patients with schizophrenia. Then, we reported the study that investigated the semantic memory structure in Japanese patients with schizophrenia by applying a newly developed data-mining technique (i.e., SVD analysis) to their category fluency data.

Semantic memory organization in patients with schizophrenia did not appear to be seriously disorganized in the 2D space representation, maintaining a similar clustering structure to that in healthy controls for highly frequent animals. However, the coherence of those animals was less salient in the 6D space, lacking pair-wise similarities to other members of the animal category. This result suggested that subtle but structural differences existed between the two groups.

### Evaluation of SVD Analysis

Although highly frequent animals were clustered in a similar manner in 2D space in patients with schizophrenia and heathy adults, the coherence of those items became weaker in 6D space in the patient group. The animal pair in the same cluster (i.e., d*og–cat, lion–tiger, elephant–giraffe*) yielded almost the same cosine values to the rest of items in healthy adults (Figure 4A). This pair-wise trend was less salient in patients with schizophrenia, except the *dog*–*cat* pair (Figure 4B). As suggested by a previous study (18), this result indicates that SVD analysis can reveal subtler structural differences in semantic memory between patients and healthy controls than are revealed by MDS or HCA.

Our results confirmed the findings from a previous study using English-speaking patients with schizophrenia (18). Thus, newly developed techniques based on a data-mining approach, such as SVD analysis, seems to be effective for elucidating the latent structure of semantic memory in patients with schizophrenia.

### Limitations

Several limitations of this study should be noted. First, we had to limit the number of items (i.e., the 20 most frequent items) due to technical reasons in creating ISM using our R program. If less frequent items were included, further differences, as reported in previous studies (18, 19), might have been observed.

Second, we did not address the issue of possible reasons for poor CFT performance in patients with schizophrenia. Some authors assume that this is due to an impoverished semantic structure (10, 40), while others explain the deterioration based on impairment of accessibility to category items (41–43). Although previous studies using SVD analysis took the latter view (18, 19), we are not certain whether semantic structure derived from SVD analysis, in which co-occurrence of items is the basic measurement, could support either the former or the latter view.

## Conclusion

The current study investigated the semantic structure of patients with schizophrenia and healthy adults by applying SVD analysis to their category fluency data. A data-mining approach, such as SVD analysis, seems to be effective for evaluating semantic memory in patients with schizophrenia, providing both a visual representation (e.g., 2D spatial representation) and an objective measure (e.g., cosine values) of the structural differences compared to healthy adults. Future studies should aim to address the mechanism of poor performance on the CFT in patients with schizophrenia, as well as the methodological problems surrounding the assessment for the deficits in semantic memory.

## Ethics Statement

The Ethical Committee of Osaka University. All participants provided written informed consents. The study protocol was approved by the Ethical Committee of Osaka University, and the procedures were conducted according to the Declaration of Helsinki.

## Author Contributions

CS designed the study, under the supervision of RH and TS. HF, HY, MF, and YY collected the data. CS conducted the analyses and wrote the initial draft. TS, FH, and RH critically revised the draft for important intellectual content. All authors contributed to the manuscript writing.

## Disclaimer

The views expressed in the submitted article are our own and do not reflect the official position of the institutions.

## Conflict of Interest Statement

The authors declare that the research was conducted in the absence of any commercial or financial relationships that could be construed as a potential conflict of interest.
